# The impact of race and age on response to neoadjuvant therapy and long-term outcomes in Black and White women with early-stage breast cancer

**DOI:** 10.1007/s10549-023-06943-x

**Published:** 2023-04-29

**Authors:** Elizabeth Terman, Jori Sheade, Fangyuan Zhao, Frederick M. Howard, Nora Jaskowiak, Jennifer Tseng, Nan Chen, Olwen Hahn, Gini Fleming, Dezheng Huo, Rita Nanda

**Affiliations:** 1 Pritzker School of Medicine, The University of Chicago, Chicago, USA; 2 Department of Medicine, Section of Hematology and Oncology, The University of Chicago, Chicago, USA; 3 Department of Public Health Sciences, The University of Chicago, Chicago, USA; 4 Department of Surgery, The University of Chicago, Chicago, USA; 5 Department of Surgery, City of Hope Orange County, Irvine, USA

**Keywords:** Disparities, Young women, Black women, Neoadjuvant chemotherapy, Pathological complete response

## Abstract

**Purpose:**

There are a paucity of data and a pressing need to evaluate response to neoadjuvant chemotherapy (NACT) and determine long-term outcomes in young Black women with early-stage breast cancer (EBC).

**Methods:**

We analyzed data from 2196 Black and White women with EBC treated at the University of Chicago over the last 2 decades. Patients were divided into groups based on race and age at diagnosis: Black women $\le$ 40 years, White women $\le$ 40 years, Black women $\ge$ 55 years, and White women $\ge$ 55 years. Pathological complete response rate (pCR) was analyzed using logistic regression. Overall survival (OS) and disease-free survival (DFS) were analyzed using Cox proportional hazard and piecewise Cox models.

**Results:**

Young Black women had the highest risk of recurrence, which was 22% higher than young White women (*p* = 0.434) and 76% higher than older Black women (*p* = 0.008). These age/racial differences in recurrence rates were not statistically significant after adjusting for subtype, stage, and grade. In terms of OS, older Black women had the worst outcome. In the 397 women receiving NACT, 47.5% of young White women achieved pCR, compared to 26.8% of young Black women (*p* = 0.012).

**Conclusions:**

Black women with EBC had significantly worse outcomes compared to White women in our cohort study. There is an urgent need to understand the disparities in outcomes between Black and White breast cancer patients, particularly in young women where the disparity in outcome is the greatest.

**Supplementary Information:**

The online version contains supplementary material available at 10.1007/s10549-023-06943-x.

## Introduction

Across the world, breast cancer is the most common malignancy diagnosed in women under 40 years of age [1, 2]. A number of studies have demonstrated that women under 40 years with breast cancer have significantly worse outcomes compared to older women. Breast cancers in young women are typically more biologically aggressive, diagnosed at a later stage, and are more likely to recur [3]. As the rate of early onset breast cancer is increasing [4] understanding this difference in outcomes is of great clinical importance. As is the case with other health measures, breast cancer incidence is impacted by race. While White women have the highest incidence of breast cancer across most age groups, in women under 35 years, Black women are significantly more likely to be diagnosed with breast cancer compared to other racial and ethnic groups [5, 6].

Not only are there differences in the incidence of breast cancer by race/ethnicity, there are consistent disparities in outcomes [7]. Minority groups—including Blacks, Hispanics, Asian Americans, and American Indians—are more likely to be diagnosed at an advanced stage as compared to Whites [5]. In comparison to all other racial/ethnic groups, Black women have the highest percentage of stage III or IV tumors at time of diagnosis [4]. Specifically with regards to triple negative breast cancer (TNBC), which is more prevalent in Black than White women, both five- and ten-year survivals are worse in Black women compared to White women [8–10]. Disparities in breast cancer outcomes by race/ethnicity are well established both nationally and within the city of Chicago [7, 11–13].

There are a paucity of data examining the potential synergistic effects of age and race on breast cancer outcomes. Specifically, data comparing responses to neoadjuvant chemotherapy (NACT) by race and age groups are lacking [14–16]. As the ability to achieve a pathological complete response (pCR) after NACT is generally associated with improved long-term outcome, evaluating pCR rates by age *and* race could provide valuable insights into mechanisms underlying cancer health disparities [14, 16–18]. Given the well-documented racial/ethnic disparities observed, along with the increasing incidence of breast cancer in women under 40—which disproportionately affects Black women—it is important to more specifically characterize breast cancer patterns and outcomes in young Black women.

In this study, we analyzed the outcomes of young Black and young White women with breast cancer treated at the University of Chicago, comparing them to each other as well as to corresponding groups of older Black and older White women. While there has not been a uniform consensus in the literature as to the specific age cutoff which defines a “young woman with breast cancer,” many studies use the age of 40 [18, 19]. As Black women and young women have poorer outcomes compared to White women and older women, we hypothesized that Black women diagnosed with breast cancer under age 40 years would have lower pCR rates and worse long-term outcomes when compared to young White women as well as older White and Black women.

## Methods

### Study design and data collection

This study utilized data from the ongoing Chicago Multiethnic Epidemiologic Cohort of Breast Cancer (ChiMEC) study that collects clinical, pathologic, and long-term outcomes of patients diagnosed with breast cancer at the University of Chicago over the last two decades [20]. 4549 patients were enrolled by the end of 2020. The study was approved by the Institutional Review Board at the University of Chicago. Male patients, non-White and non-Black patients, and patients with stage 0, stage 4, or unknown clinical stage disease were excluded from the analysis (Suppl Fig. 1). The remaining 2196 women with early-stage breast cancer in the ChiMEC study were categorized by both race and age (Suppl Fig. 1). The four focus populations were Black women $\le$ 40 years (young Black, *n* = 151), White women $\le$ 40 years (young White, *n* = 235), Black women $\ge$ 55 years (older Black, *n* = 828), and White women $\ge$ 55 years (older White, *n* = 982). A further subclassification of women receiving NACT consisted of 59 young Black women, 102 young White women, 120 older Black women, and 116 older White women (Suppl Fig. 1). Race/ethnicity in the ChiMEC study was per self-report.

### Data analysis

Patient demographics were described as mean age at diagnosis by racial and age group. Tumors were categorized as HR (hormone receptor) + /HER2 + , HR−/HER2 + , HR + /HER2−, or TNBC. In addition, clinicopathological features such as size, clinical stage, grade, and lymph node status at the time of diagnosis were collected. Tumor size between the four groups were compared with ANOVA, while the categorical features were compared via Chi-squared test. Several clinical outcomes were analyzed, including pCR (ypT0/isN0), disease-free survival (DFS), risk of recurrence, and overall survival (OS). Rates of achieving a pCR for each age/racial group and subtype were compared using a Chi-square test. The associations between both age/race group and pCR rate and age/race group and clinical trial participation rate were analyzed with a logistic regression controlling for subtype of cancer, stage at diagnosis, and tumor grade and reported as odds ratios and 95% confidence intervals [17]. Kaplan–Meier survival curves were generated for OS and DFS. A univariable Cox analysis was done comparing OS and DFS for race/age groups who received NACT, followed by a multivariable analysis adjusting for subtype, stage, grade, and Charlson Comorbidity Index [21]. Results were reported as hazard ratios and 95% confidence intervals. A piecewise Cox model was used to analyze survival outcomes in the full cohort, with results reported as separate hazard ratios for the first five years from diagnosis and beyond five years. Risk of recurrence was examined using the method by Fine and Gray, accounting for competing risk from other causes of deaths [22]. *p*-values less than 0.05 were considered statistically significant. STATA 16.1 (College Station, Texas) was used to perform all statistical analysis.

## Results

### Clinical and pathologic features

A total of 2196 women with early breast cancer identified from the ChiMEC database were included in this analysis; median follow up was 81 months. The median age of diagnosis (years) was 34.5 for young Black women, 35.3 for young White women, 65.9 for older White women, and 68.8 for older Black women (Table 1). Young women of both races trended towards diagnosis at a later stage compared to their older counterparts; young Black women had the highest incidence of stage 3 breast cancer (29.8%), compared to 16.6% of young White, 12.2% of older Black women, and 10.1% of older White women (*p* < 0.001). Young women of both races had higher rates of grade 3 tumors and more extensive lymph node involvement, with young Black women having the highest incidence of both measures. Black women had a higher rate of TNBC than their White counterparts, with young Black women having the highest rate overall (38.0%). Similar clinical and pathologic features were analyzed for the subset of women receiving NACT (Table 2).

**Table 1:** Clinical and pathologic features by racial/age group

	Young Black	Young White	Older Black	Older White	*p*-value†
	*N* = 151	*N* = 235	*N* = 828	*N* = 982	
Age at diagnosis (years)	34.5 (4.8)	35.3 (4.0)	68.8 (8.8)	65.9 (8.1)	NR
Tumor size (mm)	36.3 (26.7)	30.4 (24.4)	23.3 (21.2)	21.8 (19.8)	< 0.001*
Lymph node status					< 0.001
0	56 (45.5%)	123 (59.4%)	482 (69.1%)	599 (69.7%)	
1–3	50 (40.7%)	53 (25.6%)	153 (21.9%)	186 (21.7%)	
4–9	9 (7.3%)	24 (11.6%)	43 (6.2%)	51 (5.9%)	
10 +	8 (6.5%)	7 (3.4%)	20 (2.9%)	23 (2.7%)	
Receptor status					< 0.001
HR + /HER2−	48 (39.7%)	108 (52.2%)	471 (64.0%)	675 (76.3%)	
HR + /HER2 +	16 (13.2%)	38 (18.4%)	56 (7.6%)	72 (8.1%)	
HR−/HER2 +	11 (9.1%)	13 (6.3%)	40 (5.4%)	47 (5.3%)	
TNBC	46 (38.0%)	48 (23.2%)	169 (23.0%)	91 (10.3%)	
Stage					< 0.001
1	31 (20.5%)	84 (35.7%)	412 (49.8%)	548 (55.8%)	
2	75 (49.7%)	112 (47.7%)	315 (38.0%)	335 (34.1%)	
3	45 (29.8%)	39 (16.6%)	101 (12.2%)	99 (10.1%)	
Tumor grade					< 0.001
1	7 (5%)	17 (8%)	115 (15%)	173 (19%)	
2	42 (31%)	80 (36%)	338 (44%)	509 (55%)	
3	88 (64%)	128 (57%)	323 (42%)	242 (26%)	

Data are presented as mean (SD) for continuous measures, and *n*(%) for categorical measures

*HR* hormone receptor, *HER2* human epidermal growth factor receptor 2, *TNBC* triple negative breast cancer, *NR* not relevant

†Chi-squared test, except *ANOVA

**Table 2:** Clinical and pathologic features by racial/age group for patients receiving NACT

	Young Black	Young White	Older Black	Older White	*p*-value†
	*N* = 59	*N* = 102	*N* = 120	*N* = 116	
Age at diagnosis	34.0 (4.9)	34.9 (3.7)	65.4 (7.7)	63.1 (5.7)	NR
Tumor size (mm)	44.6 (27.7)	49.7 (98.1)	38.4 (25.5)	37.0 (27.7)	0.31*
Lymph node status					0.017
0	23 (46.0%)	54 (63.5%)	74 (69.2%)	60 (59.4%)	
1–3	20 (40.0%)	16 (18.8%)	20 (18.7%)	20 (19.8%)	
4–9	2 (4.0%)	12 (14.1%)	10 (9.3%)	15 (14.9%)	
10 +	5 (10.0%)	3 (3.5%)	3 (2.8%)	6 (5.9%)	
Receptor status					0.002
HR + /HER2−	22 (37.9%)	33 (32.7%)	24 (20.5%)	36 (31.9%)	
HR + /HER2 +	15 (25.9%)	24 (23.8%)	22 (18.8%)	28 (24.8%)	
HR−/HER2 +	5 (8.6%)	6 (5.9%)	11 (9.4%)	20 (17.7%)	
Triple negative	16 (27.6%)	38 (37.6%)	60 (51.3%)	29 (25.7%)	
Stage					0.22
1	5 (9.5%)	22 (21.6%)	17 (14.2%)	20 (17.2%)	
2	32 (54.2%)	58 (56.9%)	72 (60.0%)	64 (55.2%)	
3	22 (37.3%)	22 (21.6%)	31 (25.8%)	32 (27.6%)	
Tumor grade					0.25
1	2 (4%)	1 (1%)	1 (1%)	3 (3%)	
2	13 (23%)	19 (19%)	15 (13%)	27 (25%)	
3	42 (74%)	78 (80%)	98 (86%)	80 (73%)	

Data are presented as mean (SD) for continuous measures, and *n*(%) for categorical measures

*HR* hormone receptor, *HER2* human epidermal growth factor receptor 2, *TNBC* triple negative breast cancer, *NR* not relevant

†Chi-squared test, except *ANOVA

### Women receiving neoadjuvant chemotherapy (NACT)

#### pCR rates

**Fig. 1:**
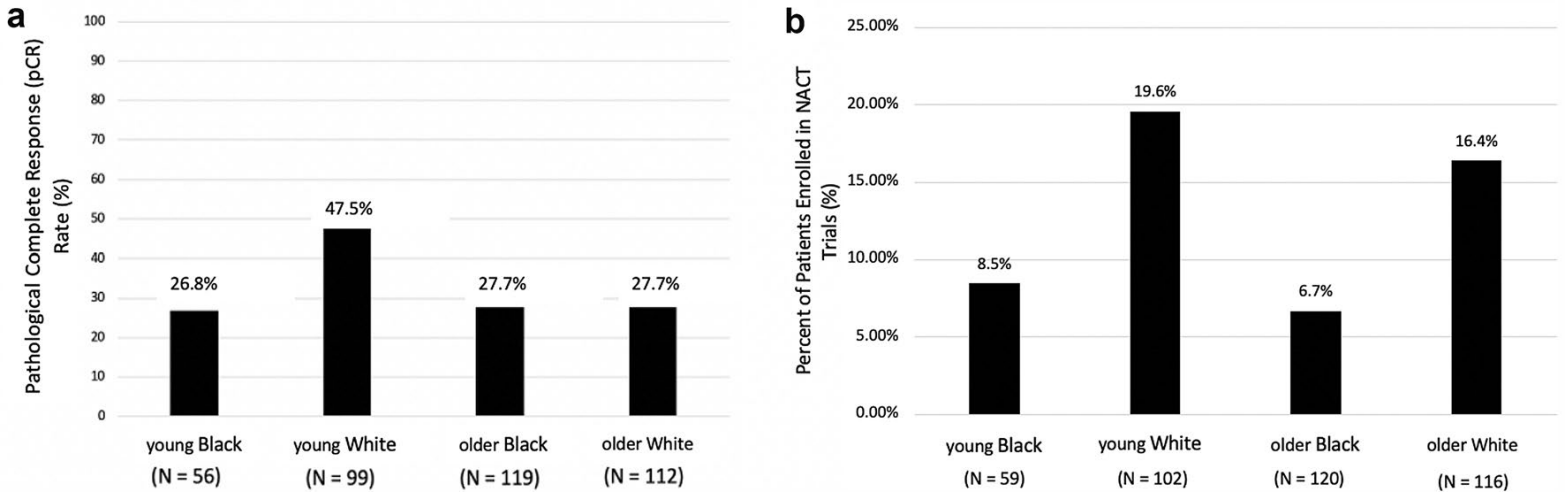
**a** Graph of pathological complete response rate amongst women receiving neoadjuvant chemotherapy between age and racial groups, *p* = 0.004 (Chi-squared test). **b** Graph of rates of trial enrollment amongst women receiving neoadjuvant chemotherapy between age and racial groups, *p* = 0.039 (Chi-squared test)

In 397 women with early breast cancer who received NACT, 26.8% of young Black women achieved a pCR, compared to 47.5% of young White women, 27.7% of older Black women, and 27.7% of older White women (*p* = 0.004) (Fig. 1A). After adjusting for subtype, grade, and stage, young Black women were still less likely to achieve a pCR as compared to young White women (adjusted odds ratio: 0.41, 95% CI 0.19–0.88, *p* = 0.022) (Suppl Table 1). Young White women were significantly more likely to achieve a pCR as compared to their older counterparts when controlling for subtype, stage, and grade (adjusted odds ratio: 3.13, 95% CI 1.66–5.89, *p* < 0.001). There was no statistically significant difference in the odds of achieving pCR in a multivariable analysis model between older Black, older White, and younger Black women (Suppl Table 1). pCR rates were further analyzed by subtype within age and racial groups (Suppl Fig. S2). Young White women consistently had the highest rate of pCR between the four age/racial groups, with 35.5% in HR + /HER2− disease (*n* = 31), 58.3% in HR + /HER2 + disease (*n* = 24), 83.3% in HR-/HER2 + disease (*n* = 6), and 45.9% in TNBC (*n* = 37). In contrast, only 20.0%, 20.0%, 60.0%, and 33.3% of young Black women achieved a pCR for these same breast cancer subtypes (*n* = 20, 15, 5, 15, respectively). Older White and Black women had similar pCR rates to young Black women (Suppl Fig. 2).

#### DFS and OS

Among women treated with NACT, young Black women had the worst DFS, followed closely by older Black women. The 10-year DFS rates for women receiving NACT were: 56.1% for young Black women, 81.7% for young White women, 50.8% for older Black women, and 74.5% for older White women. These differences in DFS amongst women receiving NACT were statistically significant (*p* = 0.0098, Suppl Fig. S3b). Before adjusting for stage, grade, subtype, and comorbidities, young Black women had a significantly increased hazard of death/recurrence compared to their young White peers (unadjusted hazard ratio: 2.19, 95% CI 1.11–4.32, *p* = 0.023). Similarly, older Black women had worse DFS than their older White peers (unadjusted hazard ratio: 1.90, 95% CI 1.10–3.28, *p* = 0.020). These differences were not statistically significant in the multivariable model (Suppl Table 2).

As with DFS, there was a marked difference in the 10-year OS rates of White and Black women receiving NACT, with rates being 71.4% in young Black women, 88.6% in young White women, 54.2% for older Black women, and 85.9% for older White women (*p* = 0.0043, Suppl Fig. S3a). Young Black women had worse OS compared to their young White counterparts (unadjusted hazard ratio: 2.22, 95% CI 0.94–5.29, *p* = 0.070; adjusted hazard ratio: 1.56, 95% CI 0.57–4.26, *p* = 0.386) (Suppl Table 2). Similarly, older Black women receiving NACT had worse OS than older White women (adjusted hazard ratio: 2.31, 95% CI 1.10–4.82, *p* = 0.026) (Suppl Table 2).

#### NACT clinical trial enrollment

We examined pCR rates in our young/old, Black/White cohorts based on clinical trial participation. Of the 397 women who received NACT, 53 participated in therapeutic trials. Young White women had the highest percent enrollment in a NACT trial, at 19.6%. 16.4% of older White women participated in a NACT trial, while 8.5% of young Black women did, and only 6.7% of older Black women were involved in a NACT trial (Fig. 1b). Young Black women receiving NACT were less likely to enroll in a trial compared to young White women (unadjusted odds ratio: 0.43, 95% CI 0.15–1.21, *p* = 0.111) (Suppl Table 3). Similarly, older Black women were less likely to enroll in a trial compared to young White women (unadjusted odds ratio: 0.34, 95% CI 0.14–0.80, *p* = 0.013) (Suppl Table 3). Neither of these differences were statistically significant after adjusting for subtype, stage, grade, and comorbidities.

Interestingly, there was no statistically significant difference in the odds of achieving a pCR based on whether or not a patient was enrolled in a clinical trial (unadjusted odds ratio: 1.00, 95% CI 0.53–1.90, *p* = 0.991). But, there was an improvement in OS (*p* = 0.036) and DFS (*p* = 0.022) for women who participated in a clinical trial (Suppl Fig. S4).

### All women with early-stage breast cancer

#### Disease-free survival (DFS)

**Fig. 2:**
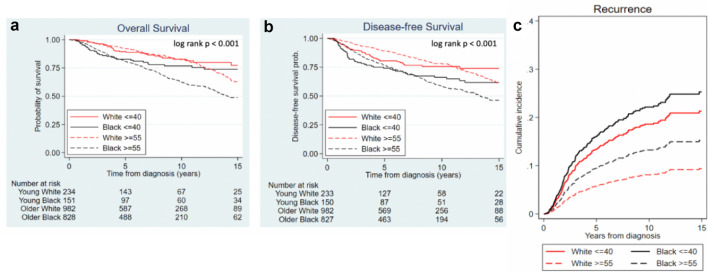
Kaplan–Meier survival curves of **a** overall survival and **b** disease-free survival by race and age, **c** graph of cumulative incidence of recurrence of the different racial/age groups

Looking at all of the women with early-stage breast cancer, regardless of treatment regimen, the 5-year DFS rate was 74.2% for young Black women, and for young White women was 80.5%. The 5-year DFS rate for older White women was 89.2%, while that of older Black women was 76.7% (*p* < 0.001, Fig. 2b). Similar trends were observed in 10-year DFS rates (*p* < 0.001, Fig. 2b). There was no statistically significant difference in DFS between young White and young Black women either before or after the 5-year stratification (adjusted hazard ratio ≤ 5 years: 1.11, 95% CI 0.64–1.94, *p* = 0.710; adjusted hazard ratio > 5 years: 1.53, 95% CI 0.51–4.58, *p* = 0.450) (Table 3). Older Black women had worse DFS outcomes compared to older White women in the first five years (adjusted hazard ratio ≤ 5 years: 1.95, 95% CI 1.45–2.62, *p* < 0.001) (Table 3). Past 5 years, there was no statistically significant difference in DFS between older White and older Back women.

**Table 3:** Association between race and age and disease-free and overall survival

Disease-free survival											Overall survival							
	Time ≤ 5 years				Time > 5 years						Time ≤ 5 years				Time > 5 years			
	Univariable analysis		Multivariable analysis^a^		Univariable analysis				Multivariable analysis^a^		Univariable analysis		Multivariable analysis^a^		Univariable analysis		Multivariable analysis^a^	
	HR (95% CI)	*p*-value	HR (95% CI)	*p*-value	HR (95% CI)		*p*-value		HR (95% CI)	*p*-value	HR (95% CI)	*p*-value	HR (95% CI)	*p*-value	HR (95% CI)	*p*-value	HR (95% CI)	*p*-value
Young Black vs. young White	1.48 (0.94–2.32)	0.088	1.11 (0.64–1.94)	0.710	1.42 (0.60–3.37)		0.428		1.53 (0.51–4.58)	0.450	1.86 (1.04–3.35)	0.037	1.69 (0.82–3.48)	0.152	0.83 (0.36–1.91)	0.654	0.60 (0.19–1.90)	0.384
Young Black vs. older Black	1.23 (0.86–1.76)	0.264	0.91 (0.57–1.45)	0.690	0.33 (0.18–0.60)		< 0.001		0.32 (0.16–0.64)	0.001	0.95 (0.62–1.46)	0.817	0.88 (0.51–1.51)	0.643	0.23 (0.12–0.44)	< 0.001	0.17 (0.07–0.41)	< 0.001
Young White vs. older White	1.94 (1.34–2.82)	< 0.001	1.60 (1.03–2.48)	0.037	0.36 (0.18–0.71)		0.003		0.25 (0.10–0.61)	0.002	1.33 (0.81–2.19)	0.251	1.08 (0.60–1.98)	0.791	0.44 (0.24–0.80)	0.007	0.33 (0.15–0.73)	0.006
Older Black vs. older White	2.34 (1.81–3.02)	< 0.001	1.95 (1.45–2.62)	< 0.001	1.53 (1.19–2.00)		0.001		1.19 (0.88–1.62)	0.267	2.62 (1.95–3.52)	< 0.001	2.09 (1.48–2.94)	< 0.001	1.58 (1.22–2.05)	0.001	1.20 (0.88–1.64)	0.244

*95% CI* 95% confidence interval

^a^Adjusted for stage, grade, subtype, and Charlson Comorbidity Index

Of the four groups, young Black women had the highest risk of recurrence, which was 22% higher than young White women (*p* = 0.434) and 76% higher than older Black women (*p* = 0.008). These age/racial differences in recurrence rates were not statistically significant after adjusting for subtype, stage, and grade (Table 4, Fig. 2c).

**Table 4:** Association between race and age and risk of recurrence

	Univariable analysis		Multivariable analysis^a^	
	HR (95% CI)	*p*-value	HR (95% CI)	*p*-value
Young Black vs. young White	1.22 (0.74–1.99)	0.434	0.97 (0.53–1.78)	0.932
Young Black vs. older Black	1.76 (1.16–2.67)	0.008	1.11 (0.66–1.86)	0.690
Young White vs. older White	2.44 (1.62–3.67)	< 0.001	1.51 (0.92–2.47)	0.098
Older Black vs. older White	1.68 (1.23–2.31)	0.001	1.33 (0.02–1.91)	0.130

*95% CI* 95% confidence interval

^a^Adjusted for stage, grade, and subtype

#### Overall survival (OS)

In terms of OS, older Black women had the worst outcomes. The 5-year OS rate was 82.7% for young Black women, 89.4% for young White women, 92.1% for older White women, and 80.8% for older Black women (*p* < 0.001, Fig. 2a). The 10-year OS rate was 76.8% for young Black women, 82.7% for young White women, 82.4% for older White women, and 62.3% for older Black women (*p* < 0.001, Fig. 2a). There was no statistically significant difference in OS between young Black and young White women over the full follow-up period. Young Black women did have improved OS compared to their older counterparts past 5 years (adjusted hazard ratio > 5 years: 0.17, 95% CI 0.07–0.41, *p* < 0.001) (Table 3). However, this difference was not significant in the first 5-year interval (Table 3). After adjusting for stage, grade, subtype and comorbidities, increased mortality in older Black vs. older White women persisted for the first five years (adjusted hazard ratio ≤ 5 years: 2.09, 95% CI 1.48–2.49, *p* < 0.001) (Table 3). This difference in OS was not statistically significant beyond five years of follow up.

#### Clinical trial enrollment

Once again, we evaluated how involvement in clinical trials may correlate with clinical outcomes. 2230 women in our cohort study had information about trial participation. Within this group, young White women had the highest percent involvement in clinical trials, at 12.4%. Older White women had the next highest percentage of patients involved in trials, at 6.1%. Young Black women had a 5.3% involvement in trials, while older Black women had a 4.2% involvement in trials (*p* < 0.050, Chi squared test) (Suppl Fig. S5). After adjusting for subtype, stage, and grade, young Black and older Black women were still less likely to be included in a trial compared to young White women (adjusted odds ratio: 0.34, 95% CI 0.13–0.90, *p* = 0.030; adjusted odds ratio: 0.53, 95% CI 0.29–0.99, *p* = 0.045 respectively) (Suppl Table 4).

Women who were enrolled in a therapeutic trial had improved OS compared to those who were not (*p* = 0.002) (Suppl Fig. S6). Whether or not a patient participated in a trial was not a significant determinate of DFS (*p* = 0.088) (Suppl Fig. S6).

## Discussion

These data reinforce the importance of focusing attention and resources on the management of young Black women with breast cancer. In our cohort, young Black women did not respond as well to NACT as their young White counterparts, had a higher risk of recurrence, and a poorer DFS; this disparity in outcome persisted, even when adjusting for grade, stage, and breast cancer subtype. We also found that young Black women who did not have a pCR tended to fare worse than young White women who did not have a pCR, and work to identify the root causes of this difference in outcomes is ongoing. Breast cancer patients who participated in clinical trials had better long-term outcomes, and Black women in our cohort had lower rates of participation in therapeutic clinical trials. While it is possible that participation in trials of novel agents lead to improved outcomes, it is more likely that trial enrollment served as a surrogate for social determinants of health, which have previously been shown to correlate with improved outcomes after a diagnosis of breast cancer [23].

It has been established that there are racial differences in engagement in screening mammography. Black women are screened less frequently than White women for a number of reasons, including limited access to care [24, 25]. Interestingly, we did not see significant differences in tumor size, nodal status, or stage at presentation in our older patients, regardless of race. In our younger population, we did observe that our young Black patients were more likely to present with higher stage disease than our young White patients, and as these women are under 40 years, this difference is unlikely to be related to differences in screening. Possibilities for this difference could be due to differences in tumor biology (Black women are more likely to have TNBC, which is faster growing and more likely to present with nodal involvement), access to care, decreased awareness of cancer risk, delay in referral to cancer providers, and/or distrust of the medical system.

However, as access to genetic testing becomes more available and screening MRIs are more frequently performed in those at increased risk, it becomes even more important to consider inequities in screening in younger women. Disparities in screening in younger women are likely to play a greater role in timing of presentation, stage at diagnosis, and eventual outcomes of women under 40 in the future.

Adherence to hormonal therapy, chemotherapy, and/or radiation may also differ based on race and age, and could in part have contributed to the disparities observed within our cohort. Across all ages, Black women more frequently report nonadherence to endocrine therapy due to increased side effects, inability to pay for therapy, and incomplete understanding of recurrence risk [26, 27]. It has also been shown that Black women are less likely to complete a full chemotherapy regimen compared to their White counterparts [28, 29]. As a whole, underserved populations are more likely to state barriers to adhering to therapy, and these issues likely contribute to the disparities in outcomes between Black and White patients, regardless of age.

Another important factor to consider is delay between diagnosis and treatment initiation. The young Black women in our cohort were more likely to have a larger gap between diagnosis and initiation of NACT than young White women; mean gap from diagnosis to treatment start was 41 days in young Black women compared to 33 days in young White women (*p* = 0.004). A preliminary investigation into the reason for such delays included psychosocial factors such as insurance issues, psychiatric illness management, and relocation for treatment. Studies have shown that patients who have longer delays in treatment, for example a greater than 8 week period between diagnosis and initiation of NACT, are less likely to achieve a pCR compared to patients without such delays [30, 31]. Rather than the delays themselves causing disparities in outcomes, it is likely that these delays serve as a surrogate marker for the underlying social determinants of health that are truly driving these differences in outcomes.

Work is ongoing to further elucidate the underlying etiology of the disparities seen in our University of Chicago cohort, with a focus on young Black women. A more comprehensive and detailed investigation is warranted in an effort to improve response and survival in this population of young women. In the meantime, it is critical to increase awareness of the poor outcomes observed in this group of particularly vulnerable women.

This study is not without its limitations. As an academic, single-center study, there is a limit to the generalizability of the results. In addition, there was a relatively small number of women receiving neoadjuvant chemotherapy, at just 397 women within the age and race parameters of this study. This translated to a low sample size when looking at pCR rates, particularly when further broken down by breast cancer subtype. A more robust analysis in a larger sample of women from multiple centers is needed to further validate these findings.

Future work will investigate the biological and social factors underlying the disparities identified in this cohort study, focusing on social determinants of health, which are likely driving the poor outcomes among young Black women. Only with a better understanding of the factors driving poor outcomes among young Black women with breast cancer can we even begin to eradicate the disparities in our most vulnerable breast cancer population.

## Supplementary Information

Below is the link to the electronic supplementary material.

Supplementary file1 (PDF 599 kb)

Supplementary file2 (DOCX 21 kb)

## Data Availability

The datasets generated during and/or analyzed during this current study are available from the corresponding author on reasonable request.
