# Supplementary material for: The impact of race and age on response to neoadjuvant therapy and long-term outcomes in Black and White women with early-stage breast cancer
Source: Breast Cancer Res Treat. 2023 Apr 29;200(1):75–83. doi: 10.1007/s10549-023-06943-x (PMC10224832; doi:10.1007/s10549-023-06943-x)
Supplement: Supplementary file 1 — Supplementary file1 (PDF 599 kb) [file 10549_2023_6943_MOESM1_ESM.pdf]

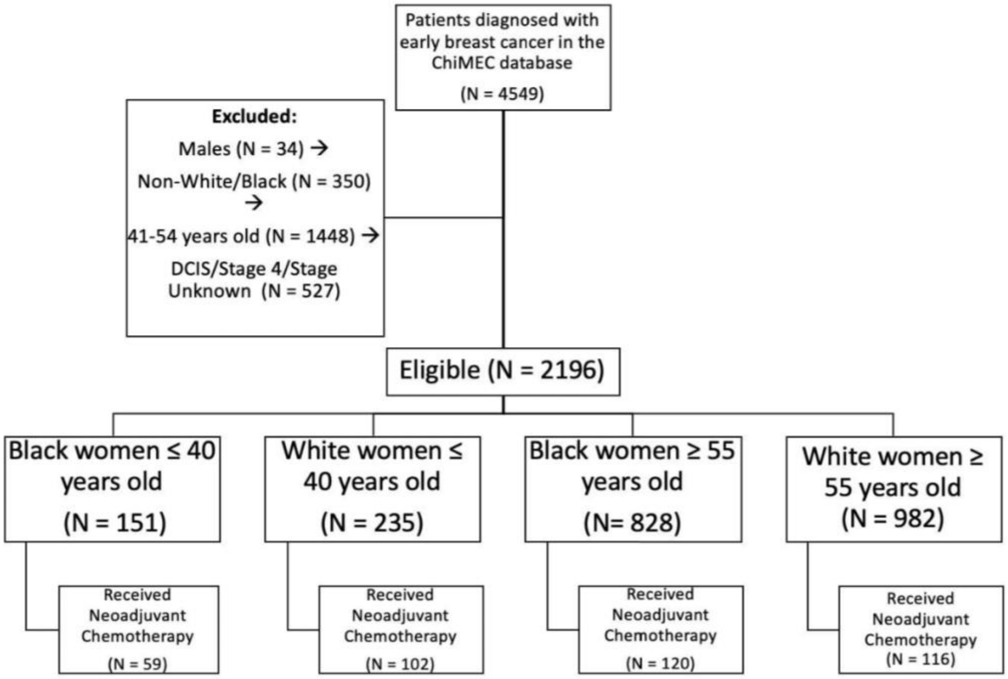

Suppl Fig. S1 A Consolidated Standards of Reporting Trials (CONSORT) diagram showing exclusion criteria and the four focus populations of the study

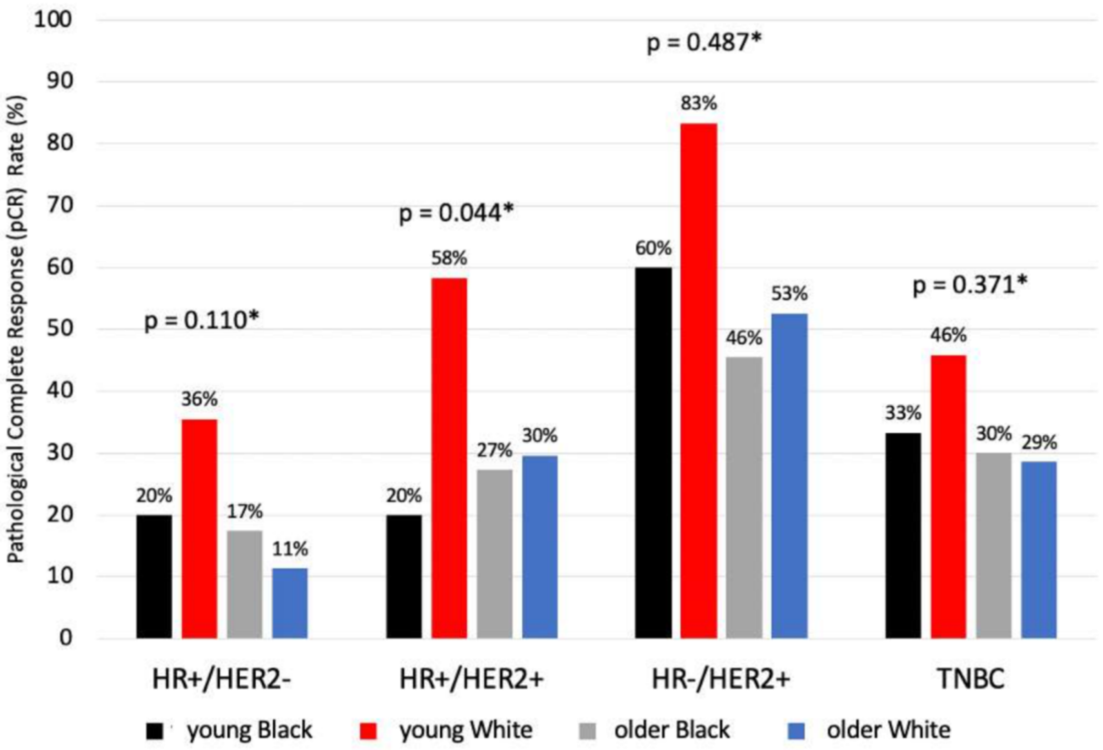

Suppl Fig. S2 Graph of pathological complete response rate amongst women receiving neoadjuvant chemotherapy stratified by subtype of breast cancer and age/racial groups

Abbreviations: pCR, pathological complete response; HER2, human epidermal growth factor receptor 2; HR, hormone receptor; TNBC, triple negative breast cancer

*p values from Chi-Squared test comparing age/racial groups within subtypes

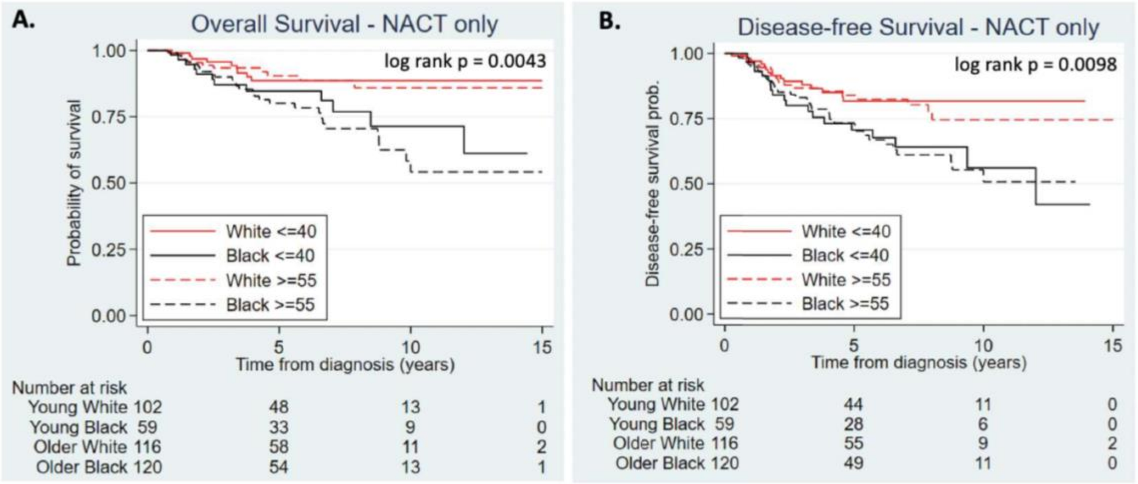

Suppl Fig. S3 Kaplan-Meier survival curves of **a)** overall survival and **b)** disease-free survival by race and age in patients receiving neoadjuvant chemotherapy

Abbreviations: NACT, neoadjuvant chemotherapy

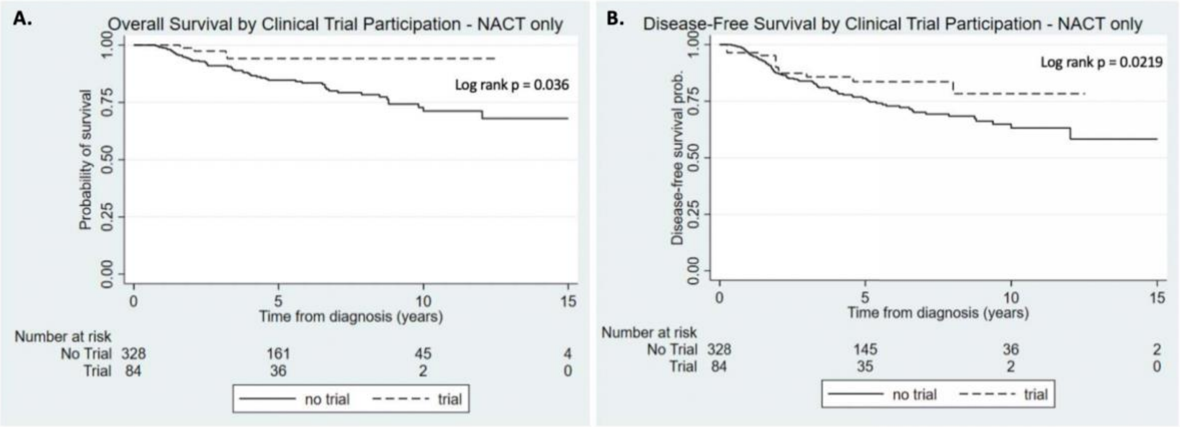

Suppl Fig. S4 Kaplan-Meier survival curves of **a)** overall survival and **b)** disease-free survival by clinical trial involvement for patients receiving neoadjuvant chemotherapy

Abbreviations: NACT, neoadjuvant chemotherapy

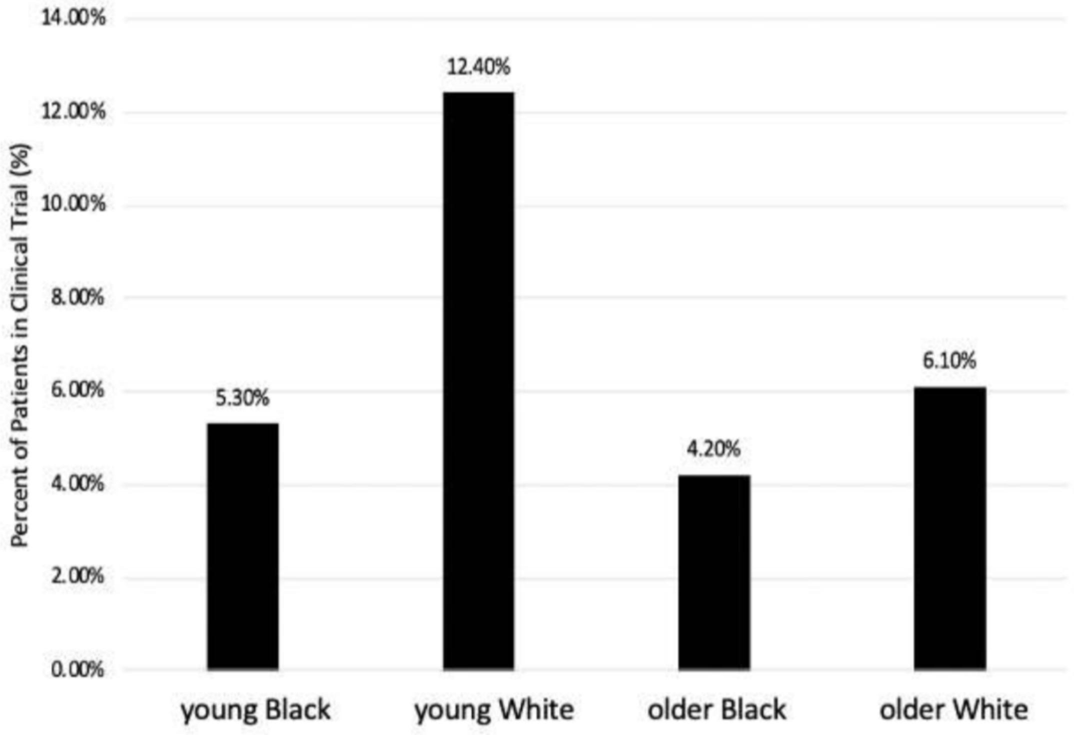

Suppl Fig. S5 Graph of rates of trial enrollment amongst all women with early-stage cancer between age and racial groups, P<0.05 (Chi-squared test)

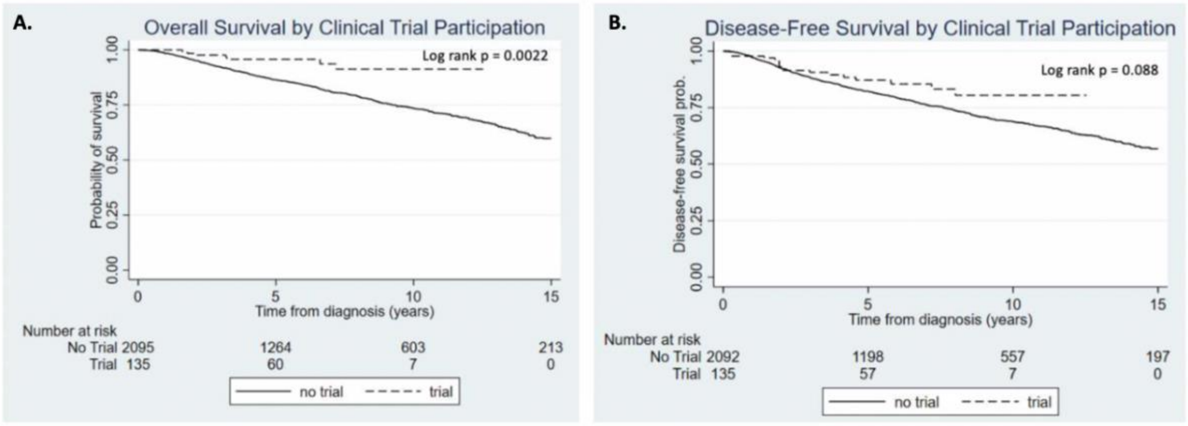

Suppl Fig. S6 Kaplan-Meier survival curves of **a)** overall survival and **b)** disease-free survival by clinical trial involvement
