# Supplementary material for: The impact of race and age on response to neoadjuvant therapy and long-term outcomes in Black and White women with early-stage breast cancer
Source: Breast Cancer Res Treat. 2023 Apr 29;200(1):75–83. doi: 10.1007/s10549-023-06943-x (PMC10224832; doi:10.1007/s10549-023-06943-x)
Supplement: Supplementary file 2 — Supplementary file2 (DOCX 21 kb) [file 10549_2023_6943_MOESM2_ESM.docx]

| **Suppl Table 1. Association of Race and Age with Pathological Complete Response** | | | | |
| --- | --- | --- | --- | --- |
|  | Pathological Complete Response (pCR) | | | |
|  | **Univariable Analysis** | | **Multivariable Analysis*** | |
|  | OR (95% CI) | P-value | OR (95% CI) | P-value |
| **young Black vs. young White** | 0.40 (0.20-0.82) | 0.013 | 0.41 (0.19-0.88) | 0.022 |
| **young Black vs. older Black** | 0.95 (0.47-1.95) | 0.896 | 1.34 (0.62-2.93) | 0.459 |
| **young White vs. older White** | 2.36 (1.33-4.18) | 0.003 | 3.13 (1.66-5.89) | <0.001 |
| **older Black vs. older White** | 1.00 (0.56-1.78) | 0.993 | 0.96 (0.50-1.83) | 0.891 |

Abbreviations: 95% CI, 95% Confidence Interval

*adjusted for stage, grade, and subtype

| **Suppl Table 2. Association Between Race and Age and Disease-Free and Overall Survival for Women Treated with Neoadjuvant Chemotherapy** | | | | | | | | |
| --- | --- | --- | --- | --- | --- | --- | --- | --- |
| Disease-free Survival | | | | | Overall Survival | | | |
|  | Univariable Analysis | | Multivariable Analysis* | | Univariable Analysis | | Multivariable Analysis* | |
|  | HR (95% CI) | P-value | HR (95% CI) | P-value | HR (95% CI) | P-value | HR (95% CI) | P-value |
| **young Black vs. young White** | 2.19 (1.11-4.32) | 0.023 | 1.80 (0.83-3.91) | 0.139 | 2.22 (0.94-5.29) | 0.070 | 1.56 (0.57-4.26) | 0.386 |
| **young Black vs. older Black** | 0.99 (0.57-1.73) | 0.981 | 0.80 (0.43-1.51) | 0.498 | 0.78 (0.40-1.52) | 0.463 | 0.66 (0.31-1.40) | 0.280 |
| **young White vs. older White** | 0.86 (0.44-1.68) | 0.663 | 0.73 (0.35-1.54) | 0.414 | 0.92 (0.38-2.21) | 0.845 | 0.92 (0.36-2.61) | 0.957 |
| **older Black vs. older White** | 1.90 (1.10-3.28) | 0.020 | 1.64 (0.92-2.93) | 0.096 | 2.62 (1.31-5.26) | 0.007 | 2.31 (1.10-4.82) | 0.026 |

Abbreviations: 95% CI, 95% Confidence Interval

*adjusted for stage, grade, subtype, and Charlson Comorbidity Index

| **Suppl Table 3. Neoadjuvant Chemotherapy Trial Enrollment by Race and Age** | | | | |
| --- | --- | --- | --- | --- |
|  | Odds Ratio of Enrolling in a NACT Trial Compared to Young White Women | | | |
|  | Unadjusted Model | | Adjusted Model* | |
|  | **OR (95% CI)** | **P-value** | **OR (95% CI)** | **P-value** |
| **young Black** | 0.43 (0.15-1.21) | 0.111 | 0.61 (0.17-2.16) | 0.446 |
| **older Black** | 0.34 (0.14-0.80) | 0.013 | 0.65 (0.23-1.85) | 0.229 |
| **older White** | 0.87 (0.44-1.72) | 0.682 | 1.24 (0.50-3.13) | 0.653 |

*adjusted for stage, subtype, grade, and Charlson Comorbidity Index

| **Suppl Table 4. Clinical Trial Enrollment by Race and Age** | | | | |
| --- | --- | --- | --- | --- |
|  | Odds Ratio of Enrolling in a Trial Compared to Young White Women | | | |
|  | Unadjusted Model | | Adjusted Model* | |
|  | **OR (95% CI)** | **p value** | **OR (95% CI)** | **p value** |
| **young Black** | 0.40 (0.18-0.88) | 0.024 | 0.34 (0.13-0.90) | 0.030 |
| **older Black** | 0.31 (0.19-0.52) | 0.000 | 0.53 (0.29-0.99) | 0.045 |
| **older White** | 0.46 (0.29-0.73) | 0.001 | 0.85 (0.48-1.53) | 0.595 |

*adjusted for stage, subtype, grade, and Charlson Comorbidity Index
